# PrEP user profiles, dynamics of PrEP use and follow‐up: a cohort analysis at a Belgian HIV centre (2017–2020)

**DOI:** 10.1002/jia2.25953

**Published:** 2022-06-30

**Authors:** Anke Rotsaert, Thijs Reyniers, Bart K. M. Jacobs, Thibaut Vanbaelen, Christophe Burm, Chris Kenyon, Bea Vuylsteke, Eric Florence

**Affiliations:** ^1^ Department of Public Health Institute of Tropical Medicine Antwerp Belgium; ^2^ Department of Clinical Sciences Institute of Tropical Medicine Antwerp Belgium

**Keywords:** HIV prevention, PrEP, Europe, retention, men who have sex with men, cohort studies

## Abstract

**Introduction:**

The number of individuals initiating antiretroviral pre‐exposure prophylaxis (PrEP) is increasing, but we do not fully understand who is coming forward for PrEP, how they use it and how they are followed‐up. The objective of this study was to examine PrEP user profiles, dynamics in PrEP use and follow‐up over time.

**Methods:**

We conducted a cohort analysis of longitudinally collected clinical record and questionnaire data among PrEP users at an HIV centre in Antwerp, Belgium, between June 2017 and March 2020. PrEP follow‐up and user profiles were examined using descriptive analyses and bivariate logistic regression. We compared early adopting PrEP users (started before June 2018) with late users. We also calculated the probabilities of switching between daily and on‐demand PrEP, and interruption, using a naïve estimator.

**Results and discussion:**

We included 1347 PrEP users in the analysis. After 12 months, retention in care was 72.3%. Median time between PrEP visits was 98 days (IQR 85–119 days). At screening visit, early adopting PrEP users (starting June 2017–May 2018) were significantly more likely to report one or more sexually transmitted infection in the prior 12 months, having used drugs during sex, a higher number of sexual partners and a history of paid sex and PrEP use prior to initiation, compared with PrEP users who initiated later (starting June 2018–February 2020). When taking PrEP daily, the probability of staying on daily PrEP at the next visit was 76%, while this was 73% when taking PrEP on‐demand. Those using on‐demand PrEP had a higher probability (13%) of interrupting PrEP care than daily PrEP users (7%), whereas those returning to PrEP care would mostly re‐start with on‐demand (35% vs. 13% for daily).

**Conclusions:**

The majority of PrEP users in this sample remained in care after 12 months. The probability of remaining on the same PrEP regimen at the subsequent visit was high. Though, we observed a diversity of transitions between regimens and interruptions in between visits. Our findings reaffirm the need to provide tailored PrEP services, counselling PrEP users across their life course.

## 1 INTRODUCTION

Pre‐exposure prophylaxis (PrEP) is highly efficacious for HIV prevention when used correctly during periods of risk exposure [1, 2]. For PrEP to have the greatest impact on the HIV epidemic, it must be taken correctly and persistently by those at substantial risk for HIV. As such, optimizing PrEP interventions requires a thorough understanding of who is using PrEP, how they are taking it and how they are followed‐up.

Belgium was one of the first countries to offer both daily and on‐demand PrEP [3, 4]. Since 2017, individuals with an increased risk of acquiring HIV have had access to partially reimbursed PrEP (i.e. currently a PrEP user pays 15 Euros per 90 pills) [5]. A Belgian PrEP care trajectory starts with a screening visit in one of 12 specialized HIV centres to assess eligibility for PrEP uptake. When eligible, the individual usually receives a first PrEP prescription at the next visit. Thereafter, PrEP users are expected to visit the HIV centre every 3 months. During these follow‐up visits, PrEP users can receive a new PrEP prescription, adherence and risk‐reduction counselling and an HIV and sexually transmitted infection (STI) check‐up. This includes testing for syphilis, gonorrhoea, chlamydia and hepatitis C. At the end of 2020, an estimated 4000 PrEP starters were registered in Belgium. These were almost exclusively men (99%) [6].

The objective of this study was to assess PrEP user profiles in a large Belgian HIV centre, the dynamics of their PrEP use and follow‐up. These insights will be useful for better understanding PrEP programme implementation and to guide PrEP care optimization.

## 2 METHODS

### 2.1 Study design

This study is a cohort analysis of longitudinal routinely collected data among PrEP users from the only HIV centre in Antwerp, Belgium.

### 2.2 Data sources and extraction

For each PrEP user, we extracted, merged and pseudonymized data from clinical records and electronic questionnaires, both collected during each routine PrEP visit. We identified PrEP users as any patient with at least two registered PrEP visits at the HIV centre between 1 June 2017 (i.e. approval of reimbursed PrEP in Belgium) and 28 February 2020 (i.e. beginning of the COVID‐19 epidemic in Belgium). The questionnaire was only available in Dutch and included socio‐demographic characteristics, eligibility criteria for PrEP, sexual behaviour and PrEP use regimen of the previous 3 months. Participants needed to be 18 years or above and be able to read Dutch to complete the questionnaire.

### 2.3 Definitions, outcomes and statistical analysis

In line with the objectives, we conducted three separate analyses.

#### 2.3.1 PrEP user profiles analysis

We divided the sample of PrEP users with a completed screening visit questionnaire into two cohorts according to the date of their screening visit, that is “early” and “late” cohort. PrEP was made available and reimbursed since June 2017. Those who initiated PrEP within the first 12 months after this date were coded as “early cohort” and those after June 2018 as “late cohort.”

We conducted bivariate logistic regression analyses to examine the associations between timing of PrEP initiation (“early” vs. “late” cohort), and socio‐demographic and sexual behavioural factors and eligibility criteria for PrEP.

#### 2.3.2 Dynamics of PrEP use analysis

Based on the response options in the questionnaire, we classified PrEP use into three categories; that is “no PrEP”, “daily” and “on‐demand”. PrEP users were considered as “interrupted PrEP care” if the time between PrEP visits was longer than 6 months, or they did not have a visit after August 2019.

We described the probabilities of switching between daily and on‐demand PrEP, and interruption, using a naïve estimator that considered all pairs of subsequent visits and the associated status at each visit. PrEP users without two subsequent visits during which a questionnaire was completed were excluded from the analysis. We did not model missing information on the PrEP regimen variable as we assumed missingness was completely at random.

#### 2.3.3 Dynamics of follow‐up analysis

To describe the dynamics of PrEP follow‐up, PrEP users were classified in three follow‐up categories: “in follow‐up”, “interrupted PrEP care” or “censored”. “In follow‐up” denotes PrEP users whose subsequent PrEP visits occurred within 6 months. When there was no visit in the following 6 months, PrEP users were considered as “interrupted PrEP care,” except when database closure was within 6 months of the last visit. In that case, they were considered as “censored.”

All analyses were done using R statistical software version 4.0.2 [7].

### 2.4 Ethical approval

The study received ethical approval from the Institutional Review Board (IRB) of the Institute of Tropical Medicine, Antwerp (IRB 1256‐18 and IRB 1352‐20) and the ethics committee of the University Hospital of Antwerp (183368). PrEP users consented to complete the questionnaire.

## 3 RESULTS AND DISCUSSION

We identified 1347 PrEP users between June 2017 and end of February 2020.

### 3.1 PrEP user profiles

Almost all 1090 PrEP users with a completed screening visit questionnaire were men (99.5%) and men who have sex with men (MSM) (97.2%), with a median age of 37 years (Table 1). Most participants were born in Belgium (83.2%) and highly educated (60.7%). Among self‐identified MSM, the most frequently reported MSM‐specific PrEP eligibility criterion was unprotected anal sex in the last 6 months (80.8%).

**Table 1:** PrEP user profiles and bivariate analyses of factors associated with early versus late cohort

	All PrEP users 1 June 2017–28 Feb 2020 *N* = 1090	Early cohort 1 June 2017–31 May 2018 *N* = 431	Late cohort 1 June 2018–28 Feb 2020 *N* = 659	Bivariate analysis^a^	
	*n* (%)	*n* (%)	*n* (%)	OR (95%CI)	*p*‐value
**Socio‐demographic**					
**Median age (years) (IQR)**	37 (30–46)	37 (30–45)	37 (30–47)	0.99 (0.98–1.00)	0.131
**Sex**					
Male	1085 (99.5)	431 (100.0)	654 (99.2)	–	–
Female	4 (0.4)	0 (0.0)	4 (0.6)	–	
Missing	1 (0.1)	0 (0.0)	1 (0.2)	–	
**Country of birth**					
Belgium	907 (83.2)	365 (84.7)	542 (82.2)	Ref	
Other	165 (15.1)	61 (14.2)	104 (15.8)	0.87 (0.61–1.22)	0.43
Missing	18 (1.7)	5 (1.2)	13 (2.0)	–	
**Highest degree of education** ^b^					
Higher education	662 (60.7)	258 (59.9)	404 (61.3)	Ref	
Lower education or no degree	417 (38.3)	170 (39.4)	247 (37.5)	1.08 (0.84–1.38)	0.557
Missing	11 (1.0)	3 (0.7)	8 (1.2)	–	
**Steady partner**					
No	601 (55.1)	237 (55.0)	364 (55.2)	Ref	
Yes	478 (43.9)	192 (44.5)	286 (43.4)	1.03 (0.81–1.32)	0.807
Missing	11 (1.0)	2 (0.5)	9 (1.4)	–	
**Sexual behaviour**					
**Median number of sexual partners in the last 3 months (IQR)**	6 (4–12)	8 (4–15)	5 (3–10)	**1.01 (1.01**–**1.03)**	**<0.001**
**Sex of sexual partners in the last 3 months** ^a^, ^b^					
Male	1071 (98.3)	427 (99.1)	644 (97.7)	1.99 (0.46–13.62)^e^	0.401
Female	36 (3.3)	10 (2.3)	26 (4.0)	0.57 (0.26–1.16)^e^	0.140
Transgender	8 (0.7)	2 (0.5)	6 (0.9)	0.50 (0.07–2.19)^e^	0.401
**Paid sex in the last 3 months**					
No	1039 (95.3)	402 (93.3)	637 (96.7)	Ref	
Yes	37 (3.4)	24 (5.6)	13 (2.0)	**2.93 (1.50**–**5.98)**	**0.002**
Missing	14 (1.3)	5 (1.2)	9 (1.4)	–	
**Condom during anal sex in the last 3 months** ^f^					
Always	129 (12.6)	46 (11.1)	83 (13.6)	Ref	
Always, except with steady partner	163 (15.9)	53 (12.8)	110 (18.1)	0.87 (0.53–1.42)	0.573
Most of the time	415 (40.6)	180 (43.6)	235 (38.6)	1.38 (0.92–2.09)	0.121
Sometimes	222 (21.7)	99 (24.0)	123 (20.2)	1.45 (0.93–2.28)	0.102
Never	91 (8.9)	35 (8.5)	56 (9.2)	1.13 (0.65–1.96)	0.671
Missing	2 (0.2)	0 (0.0)	2 (0.3)	–	
**Median self‐perceived risk to acquire HIV/STIs (IQR) (scale 0–10)**	5 (3–7)	5 (3–7)	5 (3–7)	1.02 (0.97–1.08)	0.377
**Sex under influence of drugs in the last 3 months**					
No	530 (48.6)	184 (42.7)	346 (52.5)	Ref	
Yes	529 (48.5)	238 (55.2)	291 (44.2)	**1.54 (1.20**–**1.97)**	**0.001**
Missing	31 (2.8)	9 (2.1)	22 (3.3)	–	
**PrEP use in the past**					
No	990 (90.8)	376 (87.2)	614 (93.2)	Ref	
Yes	98 (9.0)	55 (12.8)	43 (6.5)	**2.09 (1.38**–**3.19)**	**0.001**
Missing	2 (0.2)	0 (0.0)	2 (0.3)	–	
**Eligibility criteria for PrEP**					
**Self‐identified risk as general eligibility criterium for PrEP**					
MSM	1060 (97.2)	424 (98.4)	636 (96.5)	Ref	
Other than MSM	22 (2.0)	5 (1.2)	17 (2.6)	0.44 (0.14–1.12)	0.110
Missing	8 (0.8)	2 (0.5)	6 (0.9)	–	
**MSM‐specific eligibility criteria for PrEP** ^a^, ^d^					
Unprotected anal sex with at least two partners in the last 6 months	857 (80.8)	346 (81.6)	511 (80.3)	1.09 (0.79–1.49)^e^	0.610
One or more STIs in the last 12 months	329 (31.0)	162 (38.2)	167 (26.3)	**1.74 (1.33**–**2.26)** ^e^	**<0.001**
Use of PEP in the last 12 months	96 (9.1)	46 (10.9)	50 (7.9)	1.43 (0.93–2.17)^e^	0.098
Use of drugs during sex	328 (30.9)	161 (38.0)	167 (26.3)	**1.72** (**1.32**–**2.24)** ^e^	**<0.001**
HIV‐positive steady partner with detectable viral load	44 (4.2)	21 (5.0)	23 (3.6)	1.39 (0.75–2.55)^e^	0.287

Note: Values in bold indicate statistically significant results.

Abbreviations: 95% CI, 95% confidence interval; IQR, interquartile range; MSM, men who have sex with men; OR, odds ratio; PEP, post‐exposure prophylaxis; STIs, sexually transmitted infections.

^a^ Missing answers were excluded from the analysis.

^b^ “Higher education”: college or university; “Lower education or no degree”: secondary education or primary education or “I don't have a degree.”

^c^ Number of answers do not equal the number of respondents as respondents could select multiple answers. The resulting percentages can, therefore, exceed 100%.

^d^ Number of respondents having had sexual partners in the last 3 months: *N* = 1079.

^e^ Reference group is the absence of particular characteristic.

^f^ Number of respondents having had anal sex in the last 3 months: *N* = 1022.

^g^ Number of MSM reporting MSM‐specific eligibility criteria: *N* = 1060.

In the bivariate analysis, early PrEP users were significantly more likely to have had a higher number of sexual partners, paid sex and sex under influence of drugs in the 3 months before the screening visit or to have used PrEP in the past, when compared with late PrEP users (Table 1). MSM PrEP users belonging to the early cohort were significantly more likely to have had one or more STIs in the 12 months preceding screening visit and to have used drugs during sex, when compared with late MSM PrEP users.

### 3.2 Dynamics of PrEP use

**Figure 1:**
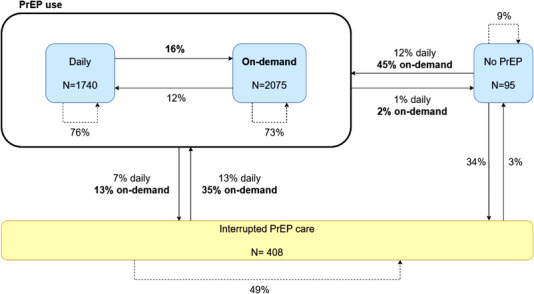
Schematic representation of probabilities to transition between no PrEP, PrEP use categories and interrupted PrEP care. *N* = total number of pairs of subsequent visits. Interrupted PrEP care includes both temporarily and final interruptions. Percentages may not add up to 100% due to rounding.

The probabilities of transitioning between no PrEP, PrEP use categories at each PrEP visit and PrEP care interruption are shown as percentages in Figure 1. A total of 4318 pairs among 907 PrEP users were included. When taking PrEP daily, the probability of continuing with daily PrEP at the following PrEP visit was 76%, while this was 73% when taking PrEP on‐demand. Daily PrEP users had a 16% probability to switch to on‐demand PrEP use. On‐demand users had a 12% probability to switch to daily PrEP use at their following PrEP visit. The probability of reporting no PrEP use was very low.

Among those who interrupted PrEP care, the probability to permanently interrupt PrEP care was 49%, whereas this was 35% to re‐start with on‐demand PrEP and only 13% to daily PrEP use.

### 3.3 Dynamics of follow‐up

Of the 1347 PrEP users, 1021 (75.8%) were in follow‐up and 326 (24.2%) had interrupted PrEP care at the end of February 2020. Retention rate at 12 and 24 months after the initial visit was 60.0% (95% CI 56.7–63.2%) and 53.6% (95% CI 48.3–58.9%), respectively. Retention at 12 and 24 months changed to 72.3% (95% CI 69.2–75.2%) and 64.5% (95% CI 59.3–69.4%), respectively, when including those PrEP users who temporarily interrupted PrEP care at 12 or 24 months, but who returned afterwards.

**Figure 2:**
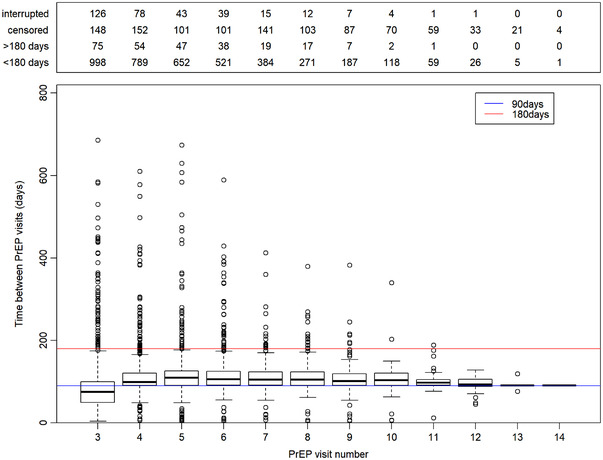
Time between PrEP visits per PrEP user. “Interrupted”: PrEP user who did not have a PrEP visit after August 2019, “censored”: PrEP user not long enough in care to decide whether PrEP user is in follow‐up or had interrupted PrEP care at this point in time, but was in follow‐up at the previous point in time, “>180 days”: time between visits exceeds 180 days, PrEP user who interrupted PrEP care, but who did return at one point “<180 days”: PrEP user in follow‐up, time between PrEP visits within 180 days.

On average, there were 4.1 and 6.6 visits since the screening visit after 12 and 24 months, respectively. The median time between follow‐up PrEP visits was 98 days (IQR 85–119 days). About 21% of the PrEP users in follow‐up at the end of the analysis (*n* = 214) had at least one time period longer than 6 months between two PrEP visits (Figure 2).

The PrEP users in this study were all considered high risk for HIV infection, as is required for PrEP reimbursement. Though, we found that early adopters reported more HIV risk behaviour as compared with PrEP users starting 1 year or later after PrEP was introduced in Belgium. The eligibility criteria for PrEP did not change during this period. We have not found other studies reporting changes over time in risk profiles of PrEP initiators. The median number of sexual partners of PrEP demonstration and open‐label study participants before enrolment is higher compared with the median number found among early adopting PrEP users in our study [8, 9, 10]. Early adopting PrEP users in our study were more likely to have more sexual partners compared with late PrEP users. This might suggest an evolution towards inclusion of lower risk profile PrEP users as the rollout of PrEP continues. Such a trend is in line with the theory of diffusion of innovations, whereby characteristics and needs of users of a new innovation might shift as implementation proceeds and becomes more common [11, 12]. However, reaching a broader public with lower HIV risk could have certain implications towards cost‐effectiveness of PrEP interventions [13, 14, 15, 16, 17]. Further research is needed to examine whether similar trends in PrEP user profiles emerge in other settings and to explore the impact of variation in HIV risk and uptake on cost‐effectiveness [13].

In Belgium, people with a migrant background, such as heterosexuals originating from sub‐Saharan African countries and non‐European MSM, may also be at high risk of acquiring HIV. However, we found very few of these individuals in our PrEP cohort [6]. Further efforts are needed to ensure every person who could benefit from PrEP has access to it.

We found that the reported PrEP regimen remained relatively steady over time, with a probability of approximately 75% to either stay with daily or on‐demand PrEP use at a subsequent visit. Nevertheless, switches between PrEP regimens and (temporarily) interruptions in follow‐up and PrEP use occurred. This is consistent with results from other studies, where 17–30% of the PrEP users switched at least once during the study period and 13–69% temporarily interrupted the use of PrEP [18, 19, 20]. To further enhance prevention‐effective PrEP adherence, healthcare providers could offer PrEP as a dynamic intervention over time, such as allowing for switching between PrEP regimens and temporarily interrupting PrEP intake. This should include adequate support in guiding PrEP users in safely starting and stopping PrEP [21].

In line with the Belgian and World Health Organization PrEP guidelines, we found a median time of 3 months between PrEP visits [22]. For half of the visits, the period in between visits ranged between 85 and 119 days. However, for a quarter of the visits, this time interval exceeded 4 months. Such irregular intervals between PrEP visits are in line with previous studies [23, 24, 25, 26]. Further research is needed to define the optimal time and modality of follow‐up according to PrEP users’ needs and risk profiles. This may include assessing who could benefit from telemedicine follow up.

An important limitation is that not all questionnaires were completed. In addition, the questionnaire was only available in Dutch. We cannot exclude that there was a self‐reporting bias in how the questionnaires were completed. This bias was, however, mitigated by using a digital tool for completing the questionnaire [27]. Responses to the questionnaires were not used by the providers to guide individual patient care. For the analysis, we only included those patients meeting our definition of a PrEP user, that is patients with at least two PrEP visits. This limits the generalizability of our findings. Secondly, we assumed that PrEP initiation coincided with a second PrEP visit since the clinical records did not contain detailed information about this event. Due to the cut‐off of our dataset period, we could have erroneously interpreted the follow‐up status of the PrEP users in our study. For example, PrEP users who received the status “interrupted PrEP care” could have scheduled a new PrEP visit after our cut‐off date.

## 4 CONCLUSIONS

While the self‐reported risks of late PrEP users still placed them at an elevated risk for HIV acquisition, this risk was lower compared with early PrEP users in our cohort. The majority of PrEP users remained in care and had a high probability of remaining on the same PrEP dosing regimen at subsequent visits. However, we observed a diverse pattern of switches between PrEP regimens and interruptions of PrEP use or care. Our findings reaffirm the need to offer PrEP services in a tailored manner, counselling PrEP users across their life course.

## COMPETING INTERESTS

The authors declare no competing interests.

## AUTHORS’ CONTRIBUTIONS

AR, TR and EF: conception of protocol, writing of the first draft of the manuscript, revision and editing of the present version of the manuscript. BKMJ: data collection, data analysis and reporting, writing of the first draft of the manuscript and revision of the present version of the manuscript. TV and CK: writing of the first draft of the manuscript, revision and editing of the present version of the manuscript. CB: data collection, data analysis and reporting. BV: global supervision of the protocol writing, writing of the first draft of the manuscript, revision and editing of the present version of the manuscript.

## FUNDING

This research was funded by Fonds Wetenschappelijk Onderzoek (FWO) Flanders as part of the research project PROMISE (“Optimise PrEP to Maximise Impact”) (S004919N).

## Data Availability

The datasets generated during and/or analysed during the current study are not publicly available, but are available upon reasonable request and if approved by the Institutional Review Board of the Institute of Tropical Medicine (Antwerp).
